# Advanced Porous Materials for Maritime Carbon Capture

**DOI:** 10.1002/adma.202515511

**Published:** 2025-12-07

**Authors:** Kelechi Festus, Ankit Mondal, Jazmine Marquez, Aishi Sikdar, Urme Podder, Qingsheng Wang, Hong‐Cai Zhou

**Affiliations:** ^1^ Department of Chemistry Texas A&M University College Station TX 77842 USA; ^2^ Artie McFerrin Department of Chemical Engineering Texas A&M University College Station TX 77842 USA

**Keywords:** carbon capture, maritime, onboard, porous materials

## Abstract

The capture and sequestration of carbon dioxide (CO_2_) has drawn significant attention due to the devastation arising from the effects of global warming. This comes from the increasing demand for the transportation of various commodities to meet the needs of the growing population of the world. Onboard capture of CO_2_ in ships has attracted much attention but suffers due to the high cost of implementation and technical setbacks. This report outlines the challenges of the current CCS technology implemented in maritime operations and evaluates porous materials (MOFs, PPNs, COFs) as a novel material class for the next generation of onboard CO_2_ capture. Engineering challenges are discussed that can create better synergies of integration in this space, and specific advanced porous materials are suggested that show great promise. If this technology can be implemented onboard vessels, it will greatly help the maritime industry adhere to internationally mandated emission regulations.

## Graphical abstract

Carbon capture from emission sources, such as marine vessels, has attracted significant attention over the years. To achieve this goal, sorbents such as metal–organic frameworks (MOFs), porous polymer networks (PPNs), covalent organic frameworks (COFs), and their post‐synthetic modifications are currently being explored. This is because of their pore tunability, high selectivity, high surface area and porosity, and fast kinetics.

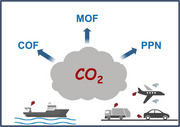

## 1 Executive Summary

The maritime sector plays a significant role in the global carbon dioxide (CO_2_) emissions cycle. It currently accounts for 3% of worldwide emissions, and with existing trends in global trade expansion, the contribution from the shipping sector is expected to rise to 18% of total emissions by 2050. The International Maritime Organization (IMO) has set benchmarks to substantially lower these figures by 2030 (20–30% reduction) and 2040 (70–80% reduction). Therefore, in order to meet these reductions within an already tight timeline, feasible carbon capture and storage (CCS) methods must be implemented on vessels sooner rather than later. Onboard carbon capture and storage (OCCS) systems using adsorption are temporary stopgaps to meet such reduction standards, especially when sorbents are solid, porous materials. Porous materials like metal‐organic frameworks (MOFs), porous polymer networks (PPNs), and covalent organic frameworks (COFs) have considerable selectivity, modularity of design, and connection to infrastructure for integration, making them viable candidates for marine OCCS applications. Yet porous‐material‐based OCCS has considerable development challenges from a technical, operational, and economic perspective. First, from a technical perspective, sorbent performance can degrade over time with the marine atmosphere—humidity and temperature shifts, scrubbing agents in seawater like sulfur oxides and nitrogen oxides—all negatively reducing capture efficiencies and application lifespans. Furthermore, sorbent regeneration requires high‐temperature thermal energy levels, which adds to the complicated energy usage management onboard. Second, from an operational perspective, space and stability constraints onboard pose tremendous integration challenges. For example, adsorption systems like temperature swing adsorption (TSA) require stable operation and reasonable adsorption capacity to effectively operate and minimize energy use, which is difficult and demanding given constant vessel movements compounded by limited space. Finally, from an economic perspective, high CAPEX and OPEX requirements for retrofitting marine vessels with specialized CCS systems render it infeasible without regulatory mandates and financial incentives for implementation. Examples of some of the best porous materials for ocean carbon capture include metal‐organic frameworks (MOFs), such as PCN‐250 and CALF‐20. For instance, PCN‐250 demonstrates a 54.2% differential in CO_2_ capture with and without relative humidity at 50%. Similarly, CALF‐20 performs great capture under humid conditions for flue gas exposure and exhibits stability up to 150 degrees Celsius. Porous polymer networks (PPNs) are also successful in marine environments; for example, PPN‐6‐CH_2_DETA has an adsorption capacity of 4.3 mmol g^−1^ at 295 K, 1 bar, exhibiting much selectivity under humid conditions. Finally, covalent organic frameworks also succeed under tests for maritime relative humidity; for example, COF‐609 has low CO_2_ performance increases of over 29% when exposed to humid conditions. Moreover, low CO_2_ concentration performance increases by 50% under humid exposure. An additional enhancement observed with these previously non‐amine functionalized material composites is the utilization of amines to elevate performance. MOFs that are functionalized with amine groups show great performance with energy‐saving capabilities of regeneration and rapid cycling for MOF amine‐modified Mg_2_(dobpdc). Furthermore, polyamine‐tethered networks such as PPN‐125‐DETA perform well over hundreds of cycles with minimal degradation, suggesting the necessity of substance viability in a maritime atmosphere. To understand the potential of OCCS technologies, additional studies must be performed to develop seawater‐compatible, resilient porous materials for anticipated service lives in actual operating conditions. Operating difficulties can be countered by hybridization or more complex cooling systems. Also, governmental policies, fiscal incentives, and supportive structures will encourage adoption. Once these interdisciplinary obstacles are overcome through extensive collaboration, OCCS technologies can transition from the realm of feasibility study to real‐world, practical application and commercialization to assist with global efforts in maritime decarbonization.

## 2 Introduction

The Global Maritime Industry is essential for international commerce, transporting over 80% of goods. However, this essential function is a major contributor to manmade greenhouse gas (GHG) emissions. According to recent findings from the International Maritime Organization (IMO), CO2 from shipping represents nearly 3% of the worldwide total, and estimates project that these emissions could increase by 50–250% by 2050 without changes to current behavior.^[^ ^1^ ^]^ With international regulatory pressures mounting—the International Maritime Organization (IMO) 2023 GHG Strategy requires compulsory emissions reductions of 20–30% by 2030 and 70–80% by 2040—the maritime industry is under pressure to decarbonize at unprecedented rates. Yet while much of the shipping industry is exploring innovative solutions—alternative fuels (ammonia, methanol), electrification, and operating solutions (slow steaming, route optimization)—one technology that has emerged as increasingly reputable is onboard carbon capture and storage (OCCS) as a feasible short‐term technological solution to afford industries time to comply with regulatory expectations sooner than later. Of the various types of OCCS available, adsorption systems using porous materials have gained attention recently due to modular technologies, high selectivity, and compatibility with existing propulsion systems.

The escalating concentration of atmospheric carbon dioxide (CO_2_) has driven a global imperative to develop efficient carbon capture technologies, with porous solid sorbents emerging as one of the most promising avenues. These materials, including metal‐organic frameworks (MOFs),^[^ ^2^, ^3^, ^4^, ^5^, ^6^, ^7^, ^8^, ^9^, ^10^, ^11^, ^12^, ^13^, ^14^, ^15^, ^16^, ^17^, ^18^, ^19^ ^]^ porous polymer networks (PPNs),^[^ ^20^, ^21^, ^22^, ^23^, ^24^, ^25^, ^26^, ^27^, ^28^, ^29^, ^30^, ^31^, ^32^, ^33^, ^34^, ^35^, ^36^, ^37^, ^38^, ^39^, ^40^, ^41^, ^42^, ^43^, ^44^, ^45^, ^46^, ^47^, ^48^, ^49^, ^50^, ^51^, ^52^, ^53^, ^54^, ^55^ ^]^ porous organic polymers (POPs),^[^ ^56^, ^57^, ^58^, ^59^, ^60^, ^61^, ^62^, ^63^, ^64^, ^65^, ^66^, ^67^, ^68^, ^69^, ^70^, ^71^, ^72^, ^73^, ^74^ ^]^ activated carbons,^[^ ^75^, ^76^, ^77^, ^78^, ^79^ ^]^ and mesoporous silicas,^[^ ^80^, ^81^ ^]^ offer high surface areas and tuneable structures ideal for selective CO_2_ adsorption. However, despite their favorable textural properties, the native performance of many sorbents often falls short under realistic operating conditions, particularly in the presence of water vapor,^[^ ^46^, ^82^, ^83^, ^84^, ^85^, ^86^, ^87^ ^]^ or at low CO_2_ partial pressures typical of post‐combustion flue gas and ambient air.^[^ ^88^, ^89^, ^90^, ^91^, ^92^ ^]^ Moisture can compete with CO_2_ for adsorption sites or even degrade sensitive frameworks, while low CO_2_ concentrations demand materials with strong binding affinity and fast kinetics to maintain efficiency. To overcome these limitations, synthetic modifications^[^ ^93^, ^94^, ^95^, ^96^, ^97^ ^]^ have been extensively explored to enhance the physicochemical interactions between sorbents and CO_2_ molecules. These strategies include post‐synthetic functionalization with amine groups,^[^ ^98^, ^99^, ^100^, ^101^, ^102^, ^103^, ^104^, ^105^, ^106^, ^107^, ^108^, ^109^, ^110^, ^111^, ^112^, ^113^, ^114^, ^115^, ^116^, ^117^, ^118^, ^119^, ^120^, ^121^, ^122^, ^123^, ^124^ ^]^ nitrogen doping, incorporation of ionic liquids, and structural tailoring to improve affinity, selectivity, and hydrophobicity. Such modifications not only elevate the sorption capacity and regeneration efficiency but also enable these materials to operate effectively under flue gas conditions, thereby advancing their viability for industrial‐scale CO_2_ capture applications.

However, the performance of sorbents under realistic conditions cannot be evaluated solely by their equilibrium CO_2_ uptake measured under dry single‐component isotherms. For maritime and other cyclic capture applications, the relevant metric is the working capacity (Δq_work_), which reflects the amount of CO_2_ that can be repeatedly adsorbed and released under humid and thermally dynamic conditions. The working capacity is defined as:

(1) $$\Delta q_{\mathrm{work}}=q_{\mathrm{ads}}\left(RH,T_{\mathrm{ads}},p_{CO_{2}}\right)-q_{\mathrm{des}}\left(RH,T_{\mathrm{des}},p_{CO_{2}}\right)$$

where q_ads_ and q_des_ are the CO_2_ loadings during the adsorption and desorption steps, respectively. This definition accounts for the effects of relative humidity, temperature, and CO_2_ partial pressure, providing a more realistic measure of a material′s cyclic performance and energy requirements for onboard capture systems.

Sorbents are generally considered to be materials that can absorb liquids or gases into their pores. They exist as solid‐based materials,^[^ ^125^ ^]^ liquid‐based materials,^[^ ^126^ ^]^ or ionic‐based materials^[^ ^127^ ^]^ and have proven to have optimized CO_2_ uptake after post‐synthetic modification. Among them, the solid‐based materials have proven to have better potency due to their inherent properties. They have higher thermal and chemical stability and faster kinetics at the gas/liquid and gas/solid interface.^[^ ^128^, ^129^, ^130^, ^131^, ^132^, ^133^, ^134^, ^135^, ^136^, ^137^, ^138^, ^139^ ^]^ They are engineered to possess pores that are sizable enough to fit the kinetic diameter of the molecules of interest in each environment. Interestingly, it has found applications in areas such as photocatalysis,^[^ ^140^, ^141^ ^]^ optoelectronics,^[^ ^142^, ^143^ ^]^ gas separation,^[^ ^144^, ^145^, ^146^, ^147^, ^148^, ^149^, ^150^, ^151^, ^152^, ^153^, ^154^, ^155^ ^]^ bioimaging,^[^ ^156^, ^157^ ^]^ conductivity,^[^ ^158^, ^159^, ^160^ ^]^ photoluminescence,^[^ ^161^, ^162^, ^163^, ^164^ ^]^ and energy storage,^[^ ^160^, ^165^, ^166^ ^]^ just to mention a few. The application of this class of materials for gas separation has been extensively studied due to the growing effects of global warming, among others. This report will discuss the process modules, current sorbents, candidate metal‐organic frameworks (MOFs), and a strategy to boost MOF affinity for CO_2_ capture in ships.

## 3 Process Models in Maritime Carbon Dioxide Capture

**Figure 1:**
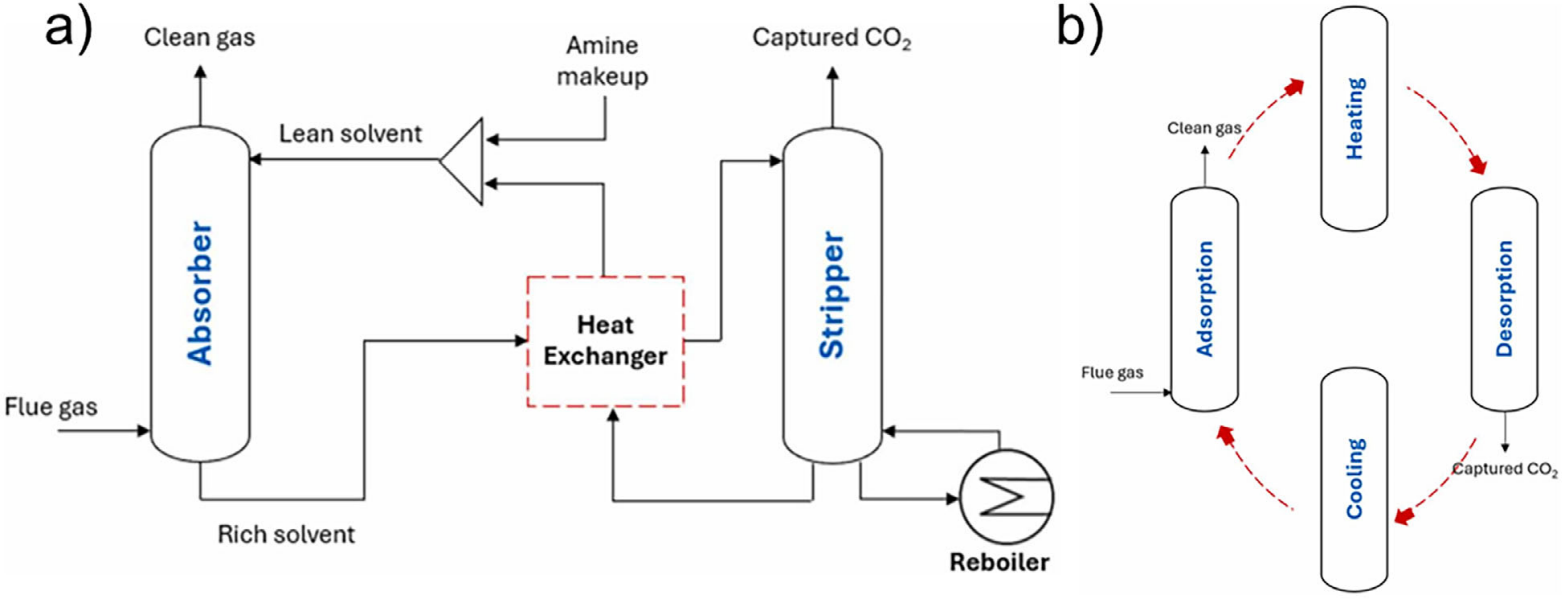
Schematic representation of (a) chemical absorption and (b) temperature swing adsorption process. Reproduced with permission.^[^ ^187^ ^]^ Copyright 2017, Elsevier.

The aim of decarbonizing the shipping industry by 2050 has steered researchers to develop and simulate processes that would help achieve this goal. Adopting post‐combustion carbon capture (PCC) for ships is feasible because of reasonable retrofitting costs and equipment design variability. There are six processes being investigated for post‐combustion carbon capture (PCC) potential onboard ships: chemical absorption, temperature swing adsorption (TSA), membrane separation, calcium looping, cryogenic separation, and integrated absorption‐mineralization. The chemical absorption process aboard maritime transportation has been widely investigated in literature due to its commercial readiness. **Figure** **1**a illustrates the absorption process used in simulating the PCC process. These simulations have shown the importance of solvent choice,^[^ ^167^, ^168^, ^169^, ^170^ ^]^ process configuration,^[^ ^171^, ^172^, ^173^, ^174^ ^]^ heat sources,^[^ ^175^, ^176^, ^177^, ^178^ ^]^ equipment design,^[^ ^179^, ^180^, ^181^, ^182^ ^]^ and fuel type^[^ ^183^ ^]^ as studied. Techno‐economic analyses have provided the potential Energy Efficiency Design Index (EEDI) for different reference vessels and the corresponding improvements and cost of carbon capture (CCC) with the addition of a PCC.^[^ ^171^, ^184^ ^]^ These simulations showed that the EEDI can be reduced by half with a retrofitted PCC, and optimizing the solvent and process configuration has helped decrease the CCC.^[^ ^167^ ^]^ These results were consistent even with the addition of a heat source or a customized process configuration. A cradle‐to‐gate life cycle analysis was also accomplished for a Sleipnir, which indicated that port and daily operations are important factors to consider when designing an efficient PCC absorption system for maritime transportation.^[^ ^185^ ^]^ The routine and motion of the ship, as well as the voyage route, affect the CO_2_ generation of the engine, which also impacts the efficiency of the PCC process. Moreover, Farooq and Karimi have developed a detailed design and costing procedure of amine‐based CO_2_ absorption PCC for LNG‐fueled ships, which can also be modified to address different ships or fuel types.^[^ ^186^ ^]^ For instance, the design of a 53,200 DWT container feeder was analyzed to develop the tonnage of CO_2_ per day that can be achieved using the volume of storage tank equation reported. It was observed that for a 90% CO_2_ recovery rate, a 3330 m^3^ tank, and a 35.33 kg s^−1^ flue gas flow rate of CO_2_ to the system, 118 t CO_2_ capture per day can be achieved. This is a crucial finding because it guides the development of storage systems or vessels for CO_2_ capture, which eventually helps to curb the amount of CO_2_ emissions. Furthermore, it provides techno‐economic direction and predictability of maximum achievable output, since, based on the equation reported, the tonnage of CO_2_ capture per day can be calculated. Hence, the chemical absorption process has demonstrated preparedness for upscale application. However, the common issue that these simulations have encountered is the insufficient flue gas heat, which makes it difficult to achieve 90% CO_2_ capture rate in addition to balancing energy efficiency, minimizing CO_2_ production, and having a suitable space requirement. Other processes are available to address this issue but have not been extensively studied for maritime onboard PCC, due to either a combination of low commercial readiness and low capture efficiency, or cost‐ and energy‐intensive operations.^[^ ^187^ ^]^ However, the continuous emergence of high‐performance adsorbents allows for the continuous improvement of the TSA process, which increases its potential as a PCC pathway.

TSA, shown in Figure 1b, addresses the concern of chemical absorption with regard to the high thermal energy required for regeneration because adsorbents can be exploited at relatively lower temperatures. In theory, TSA has a smaller space and energy requirement compared to the absorption process. However, application to onboard maritime carbon capture is deterred because of the large volume of ship exhaust emissions.^[^ ^188^ ^]^ There are different types of reference vessels with a variety of engine capacities. Relatively, a ship has a larger volume of engine exhaust because it is required to power all the ship′s activities throughout a voyage, long or short. Hence, the larger the ship, the greater the engine capacity and its emissions, leading to large or multiple adsorption towers and a huge number of adsorbents to achieve the desired CO_2_ capture rate. This is unfavorable because the input cost for the equipment is higher compared to the absorption process. Furthermore, the space requirement and retrofitting feasibility decline as the engine capacity increases. Moving forward, identifying the integration surfaces for the adsorption process is necessary to begin investigations on different process configurations and design considerations, to determine the effect on the EEDI and the equivalent CCC of the process.

## 4 Implementation Setbacks of Sorbents in Maritime Carbon Capture

Despite their potential, the implementation of porous‐based materials for CO_2_ capture on ships faces numerous setbacks that limit their widespread adoption. These challenges range from materials degradation under harsh marine conditions and energy‐intensive regeneration processes to integration difficulties due to limited onboard space and ship motion. Additionally, economic barriers, including high capital and operational expenditures, hinder the commercial viability of these systems. This section aims to critically examine these multifaceted barriers, drawing from recent experimental studies, techno‐economic models, and pilot initiatives, to propose directions for addressing these challenges.

### 4.1 Technical Setbacks: Materials and Process Challenges

The most significant issue with porous materials used in situ on vessels is the physical and chemical efficacy of the adsorbents. For example, porous materials for CO_2_ capture include amine supports, zeolites, activated carbon, and metal‐organic frameworks (MOFs). These materials have a high potential for capture in laboratory settings under varying temperature and humidity; however, porous materials exposed to maritime conditions over time, such as ship exhaust mixtures, may be diminished. For instance, according to Balsamo and coworkers, flue gases with excessive water vapor, as well as sulfur and nitrogen oxides (SO_x_/NO_x_) and particulates, may decrease adsorption potential as they occupy active sites and/or cause chemical degradation of essential functional groups.^[^ ^187^ ^]^ The flue gas in an LNG‐fuel ship, as reported by Balsamo and coworkers, has a composition of 5% CO_2_, 5% H_2_O, and a higher amount of N_2_. Due to the similarity in the volume of H_2_O present compared to CO_2_, it poses a difficulty in the application of sorbents to realize this goal. This is because water is more attracted to most available sorbents than CO_2_ and has a smaller kinetic diameter.

For example, while MOFs have remarkable surface area and tunable functionality, they become sensitive to moisture in some cases and experience structural collapse or pore blockage. While hydrophobic MOFs and post‐synthetic modifications are explored, they are still mostly at a lab level and not ready for extensive ocean‐going implementation. While functionalized carbons and supported amine materials are less susceptible to moisture‐related issues, they fall victim to oxidative degradation and fouling, needing additional regeneration or reapplied over time. In addition, while some TSA can use waste engine heat for regeneration, it still needs precise thermal management to avoid sorbent degradation and promote effective desorption, a fine‐tuning effort that is almost impossible to maintain at sea.

Moreover, to sustain non‐stop operations aboard vessels, regeneration cycles must be low‐energy and rapid. Unfortunately, many porous materials generate slowly and require higher thermal energies, especially for the TSA systems. Even the ideal sorbents indicate that desorption regenerations require temperatures higher than 100 degrees Celsius; thus, heat integration efforts are required, such as organic Rankine cycles or heat pumps which add complexity to the system and additional energy demand onboard. Ultimately, porous materials come with overly complicated thresholds that compromise performance, reliability, and durability in efficiencies that would not otherwise be known in a perfect world.^[^ ^189^ ^]^

### 4.2 Integration Constraints: Space, Motion, and Engine Compatibility

Ships—particularly not new ones and those retrofitted with CCS—are comprised of spaces and operations that do not mesh with delicate, large carbon capture storage (CCS) possibilities. For instance, porous material based CCSs that utilize TSA systems require multiple beds, large amounts of piping, supplementary systems like compressors and heat exchangers. Such ancillary equipment housed on the vessel will displace space already at a premium and dedicated to propulsion, transport, and shipping, fuel, and vital life‐support systems. Furthermore, the additional requirements for CCS will merely add unnecessary weight or stability to a system where a center of gravity has been painstakingly designed for maximum efficacy. While CCSs could be designed smaller as hollow fiber adsorbers or monolithic sorbent beds, these configurations would still compete for space as opposed to padding it out.^[^ ^1^ ^]^

Moreover, marine environments are highly dynamic. Ships experience constant vibration, roll, pitch, and yaw, all of which can disrupt fluid distribution inside adsorption columns. This results in channeling, reduced residence time, and inefficient mass transfer, key issues that degrade capture performance. Unlike land‐based installations where systems operate under stable conditions, onboard carbon capture and storage (CCS) systems must maintain performance while exposed to shocks, tilts, and operational fluctuations. Adsorption materials and column internals must be reinforced to withstand these mechanical stresses, which adds weight and complexity to the system.

Furthermore, the use of marine engines operating at consistent cruising load or, on the other hand, the capacity for high‐speed turning and resulting exhaust venting issues means that the makeup and emission of flue gases change, too. Therefore, adsorption becomes more difficult to stabilize as this requires a consistent gas emission to effectively adsorb and a timed emission for later desorption. Yet some engines (LNG dual‐fuel) possess a more consistent emissions scenario. Still, regardless of the integration of carbon capture and storage (CCS), attention must be paid to the consistent yet inconsistent dynamics projected by energy systems within the vessel, which render control and automation difficult.^[^ ^1^, ^187^, ^190^ ^]^

### 4.3 Economic Viability: CAPEX, OPEX, and Uncertain Returns

Economic considerations dominate one of the most restrictive shortcomings of porous‐material OCCS technology within the commercial maritime shipping sector. Vastly efficient maritime applications and components require anti‐corrosive capabilities and space‐saving qualities so they don′t take up valuable cargo within a vessel′s constrained, compartmentalized design. For example, from the upfront capital cost to the yearly maintenance and energy consumption requirements, adsorption‐based CCSs on vessels are several times more expensive than land‐built counterparts. In addition, vessels require thermal integration systems (e.g., WHR loops, ORCs) to enable TSA functionality, which is a tangible part of the upfront capital cost.^[^ ^191^ ^]^

When it comes to costs, Musa et al. forecast the CAPEX and OPEX of all three CC technologies—cryogenic separation, chemical absorption, and membrane separation—and evaluate the final assessment of onboard integration.^[^ ^192^ ^]^ When implemented in power plants as retrofits, chemical absorption technologies also require high CAPEX because the absorber and stripper units are costly; packed columns are the costliest parts. However, it presents trade‐offs between CAPEX and OPEX.^[^ ^175^ ^]^ Less absorber installed translates to a lower upfront cost, but a higher solvent recirculation rate and reboiler duty. This means it needs more energy, which contributes to OPEX the most. Cryogenic separation has a moderate CAPEX and a low OPEX, which means it has a high ability to reduce the cost impact. On the other hand, Chemical absorption has a high CAPEX and a moderate OPEX, which means it has a moderate ability to reduce cost impact. Membrane separation has a high CAPEX, the highest OPEX, and relatively lower ability to reduce cost impact.^[^ ^191^ ^]^ Mixed Matrix Membrane (MMM) has been explored for the capture of CO_2_ and its efficient separation from other gases such as N_2_. This usually comprises the combination of a polymer matrix and a sorbent such as MOF, COF, etc, which could be amine‐functionalized or nanosized. Sivaniah and coworkers have reported the development of an MMM from PIM‐1 and UIO‐66‐NH_2_.^[^ ^193^ ^]^ It was observed that due to the MOF′s interfacial adhesion with the polymer, which decreases the number of defects as a result of the hydrogen bonding, the flexibility of the MOF and PIM‐1 chain is reduced, thereby hindering large molecules from going through it. This new structure gave rise to an optimized selectivity for CO_2_ coupled with the amines that increased the CO_2_ affinity.

Economic modeling projects the price of carbon removal at €77 to €163 per ton of CO_2_ with standard monoethanolamine (MEA) carbon capture systems on board vessels. However, with the more novel approach of TSA, this number drops to €99 per ton—but only if the systems can be placed on existing vessels operating at sea with waste heat recovery systems—under perfect operating conditions and extended pay‐back periods. Such figures are greater than either the price of carbon trading trigger buys or negative fuel taxation reductions, meaning the installation projects would not be favorably seen by any vessel owner unless governmental subsidies were extensive or regulatory compliance was implied.^[^ ^1^ ^]^

### 4.4 Regulatory and Infrastructural Uncertainties

Yet another significant hurdle to implementation is the lack of regulatory consistency and support infrastructure relative to onboard carbon capture and storage (OCCS) technology. The International Maritime Organization (IMO) has not yet adopted any carbon capture‐related compliance into its regulatory agency measures; it has only focused on fuel‐dependent and fuel‐independent performance such as the Carbon Intensity Indicator (CII) and Energy Efficiency Design Index (EEDI). Without an established protocol for carbon capture and storage (CCS) regulations of technology approval, effectiveness verification, or emissions reduction allowances, shipowners are left in limbo as to whether their OCCS technology will be compatible or income‐generating with EU ETS or other international emissions trading systems.^[^ ^1^, ^194^ ^]^

Yet, even if onboard carbon capture and storage (OCCS) systems are feasible from a technological and financial standpoint, they rely upon an even more globalized logistical system. For something to be unloaded, stored, and transported as cargo, it needs to have an established port infrastructure. Currently, ports are not equipped to handle significant influxes of captured CO_2_. Furthermore, establishing new infrastructural projects—subsurface pipelines, liquefaction terminals, solid mineralization plants takes years of planning and execution with suggested investments of billions of dollars. Moreover, it must be a concerted international effort to create a place where potential next steps for carbon capture will be accommodated at ports with common standards of treatment; otherwise, ships will go off the coast and have no place to deposit their captured cargo. Therefore, without a logistical international system already in place, onboard carbon capture and storage (OCCS) systems might not be able to discharge at all.^[^ ^175^, ^191^ ^]^

## 5 Currently used Sorbents for Maritime Carbon Capture

Although studies involving the practical implementation of sorbents in maritime carbon capture remain relatively limited, a select number of sorbents have been investigated specifically for their applicability in shipboard environments. Studies have been conducted on the potential application of alkali metal carbonates to reduce CO_2_ emissions from marine diesel engines.^[^ ^195^, ^196^ ^]^ Balsamo et al. studied K_2_CO_3_‐based sorbents for CO_2_ capture from model marine diesel engine exhaust.^[^ ^197^ ^]^ It was reported that bulk K_2_CO_3_ yields 0.138 mmol g^−1^ capture capacity, while its alumina‐supported form achieves 43% CO_2_ conversion when tested with marine diesel engine exhaust (5% CO_2_, 5% H_2_O, 90% N_2_; temperature 60–105 °C). PPN‐6‐CH_2_TETA, a porous polymer network functionalized with an amine group, was investigated for CO_2_ capture in internal combustion engine exhaust through temperature swing adsorption.^[^ ^198^ ^]^ It operated at 30 °C for adsorption and 150 °C for desorption and achieved a CO_2_ working capacity of 0.1 kg CO_2_ per kg of adsorbent. The material shows good performance under humid conditions and can be applied to flue gases. A simulated truck‐scale plant using this sorbent achieved 90% capture efficiency with waste heat integration for regeneration. Its applicability to low‐grade heat and resistance to moisture make it well‐suited for mobile CO_2_ capture in the maritime sector. Calcium oxide (CaO) is quite a promising sorbent for marine carbon capture, applied significantly in calcium looping technology. One approach includes NaOH absorbing CO_2_ from ship emissions, which subsequently reacts with CaO to form CaCO_3_ while regenerating NaOH.^[^ ^199^ ^]^ The process reduces onboard storage needs and facilitates the potential sale of CaCO_3_. Compatibility of CaO with regeneration at high temperature and fluidized beds also supports its marine application potential. One system proposed by Bortuzzo et al. captures CO_2_ from ship exhaust using Ca(OH)_2_ and converts it into solid calcium carbonate (CaCO_3_), which can be stored or discharged into the ocean. This avoids energy‐intensive liquefaction of CO_2_, offering operational and logistical advantages over conventional CCS technologies.^[^ ^194^ ^]^

Chemical absorption using aqueous solvents like monoethanolamine, MEA, or advanced blends is the most mature onboard CCS concept. One study explored ammonia‐based carbon capture on LNG‐fueled ships, integrating hot‐side heat recovery and Exhaust Gas Recirculation (EGR) to reduce energy and costs. A validated rate‐based model was scaled up for ship flue gas, with captured CO_2_ reliquefied and stored onboard. Optimal capture occurred at 90% rate and 10 wt% ammonia, with EGR lowering capture costs by 10%.^[^ ^200^ ^]^

Porous materials such as metal–organic frameworks (MOFs), zeolites, and activated carbon have been extensively investigated for carbon dioxide capture; however, their application within the maritime sector remains relatively underexplored and limited to early‐stage research or conceptual studies. However, materials that offer high CO_2_ selectivity, stability under humid conditions, and low regeneration energy should be best suited for maritime applications. MOFs such as PCN‐250 show a higher CO_2_ uptake under humid conditions as compared to dry conditions. PCN‐250 showed a 54.2% increase in CO_2_ absorption capacity under 50% relative humidity compared to dry conditions.^[^ ^201^ ^]^ The zinc‐based MOF CALF‐20 satisfies multiple technical criteria needed for onboard carbon capture in the maritime sector, including hydrophobicity, thermal stability, low regeneration energy, and ease of processing.^[^ ^202^ ^]^ This robust material exhibits preferential CO_2_ adsorption even at 40% relative humidity and retains its adsorption performance under flue gas conditions of up to 150 °C. Furthermore, recent experiments and molecular simulations on CALF‐20 under H_2_O/CO_2_ reveal that water does not simply physisorb independently, it forms hydrogen‐bonded networks and induces guest‐dependent framework responses, with structural changes evident at ≥20% RH. Under co‐adsorption, CO_2_ and H_2_O compete for similar pore regions, and the presence of one reduces the mobility of the other, producing non‐trivial kinetics beyond dry‐gas predictions. These thermodynamic–kinetic couplings rationalize observed rate limitations under wet flue gas and imply longer adsorption steps, and reduced per‐bed productivity in TSA at constant capture.^[^ ^203^ ^]^ In a study by Kumar et al., four physisorbent porous materials – Zeolite 13X, HKUST‐1, Mg‐MOF‐74, and SIFSIX‐3‐Ni, were analyzed for their CO_2_ uptake in humid conditions.^[^ ^204^ ^]^ Mg‐MOF‐74 and HKUST‐1 suffer significant deterioration of CO_2_ uptake capacity under humid conditions, largely due to excessive water absorption and structural instability. Zeolite 13X, by contrast, remained structurally intact for 14 days but suffered low CO_2_ uptake upon exposure to humidity due to competitive water adsorption and required regeneration at high temperatures. SIFSIX‐3‐Ni demonstrated high CO_2_ selectivity, excellent humidity tolerance, and low‐energy regeneration, making it an ideal physisorbent for maritime carbon capture applications.

## 6 Potential Metal–Organic Framework for Maritime Carbon Capture

Metal‐organic frameworks (MOFs), a solid‐state sorbent material, have gained increasing interest from researchers in different fields of science. It originated from the work of Omar M. Yaghi and his team in 1995^[^ ^2^ ^]^ and 1998^[^ ^205^ ^]^ when they successfully synthesized and characterized MOF‐5, which consists of a tetrahedral [Zn_4_O]^6+^ cluster and a BDC = 1,4‐benzenedicarboxylate ligand that grew into a 3D cubic network for the first time. This achievement has given rise to the synthesis of different kinds of MOF materials for various applications over the years. The whole concept of MOF is built on reticular chemistry, which involves the bonding of molecular building blocks to fabricate a crystalline porous moiety.^[^ ^206^, ^207^ ^]^ The application of MOF for carbon dioxide capture has been extensively studied and reported.^[^ ^85^, ^208^, ^209^, ^210^, ^211^, ^212^ ^]^ While most of them are not high performing, many others possess promising optimal CO_2_ uptake potential at room temperature and hence are promising for onboard carbon capture in ships. This section will focus on robust MOFs that can be incorporated into ships for onboard carbon capture.

**Figure 2:**
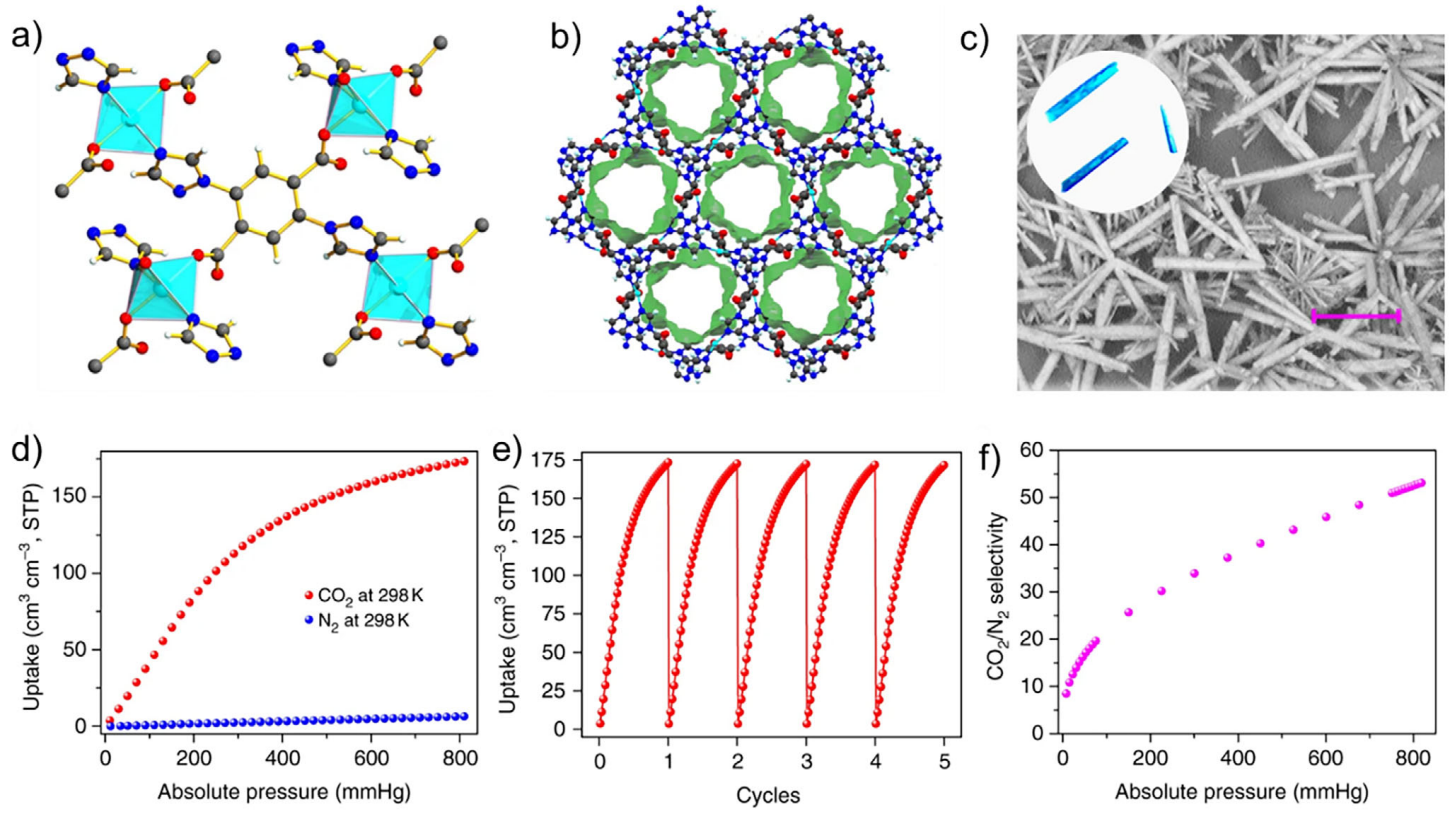
Properties of FJI‐H14. a) The coordination environment of the Cu(II) ions is a four‐connected node, and BTTA is also a four‐connected node. b) The 1D nanoporous channels along the crystallographic c direction. c) Morphology comparison between SEM image of FJI‐H14 microcrystals and of FJI‐H14 single crystals (inset). Scale bars, 10 µm. d) N_2_ and CO_2_ adsorption isotherms for FJI‐H14 at 298 K. e) Cycles of CO_2_ adsorption for FJI‐H14 at 298 K. f) CO_2_/N_2_ selectivity for the 15/85 CO_2_/N_2_ mixture at 298 K. Reproduced with permission.^[^ ^213^ ^]^ Copyright 2017, Nature Communications.

Furthermore, Liang et al reported FJI‐H14, a copper‐based MOF synthesized from 2,5‐di(1H‐1,2,4‐triazol‐1‐yl)terephthalic acid (H_2_BTTA) and Cu(NO_3_)_2_ with a gravimetric uptake of 146 cm^3^ g^−1^ at 298 K and 1 atm and thermally stable up to 230 °C.^[^ ^213^ ^]^ It has a CO_2_/N_2_ IAST selectivity of 51 and sustained its CO_2_ capacity after 5 cycles. A mechanistic study revealed the binding sites in the MOF to be open Cu(II) sites, carboxylate oxygen atoms, and a charged proton, which implies that it has unique three‐binding CO_2_ sites. The properties of this MOF (**Figure** **2**) and its scalability, and acid‐base resistance make it a good candidate for onboard carbon capture (OCC) amongst others. Yaghi, Navarro, and their team in 2024 explored the potential of MOF‐808‐Lys with a BET Surface area of 701 m^2^ g^−1^ among other MOFs for CO_2_ capture.^[^ ^214^ ^]^ Specifically, this sorbent was used for direct air capture (DAC) and demonstrated exceptional CO_2_ uptake. It was discovered that MOF‐808‐Lys has an uptake of 1.205 mmol g^−1^ at 400 ppm/50% RH (relative humidity), which exceeded the earlier report of Yaghi et al.^[^ ^215^ ^]^ The uptake was facilitated by moisture, which promoted bicarbonate formation through CO_2_ binding to the amine sites in the framework. MOF‐808‐Lys is composed of Zr_6_O_4_(OH)_4_ metal nodes, BTC (benzene‐1,3,5‐tricarboxylic acid) linker, and L‐lysine coordinated to the framework via carboxylate (Figure 2). A dynamic breakthrough experiment was used to confirm performance, and the average working capacity was 0.696 mmol g^−1^ per cycle, without any degradation of the MOF structure and CO_2_ uptake capability observed. Li et al. used a self‐supporting foam strategy to design HK@BNNS‐PEI for CO_2_ capture.^[^ ^216^ ^]^ In this work, a series of sorbents (HK@BNNS‐PEI, MIL@BNNS‐PEI, ZIF@BNNS‐PEI) were made from HKUST‐1, MIL‐100(Fe), and ZIF‐8. Among them, HK@BNNS‐PEI had the best performance. It has a CO_2_ uptake at 1 bar and 298K of 4.47 mmol g^−1^ and a 5 times desorption rate compared to pristine HKUST, which only had a CO_2_ uptake of 3.15 mmol g^−1^ (at 1 bar and 298K). This is because the BNNS (Boron Nitride Nanosheets) acts as the heat conduction network while PEI (Polyethyleneimine) reduces interfacial resistance and provides more binding sites for CO_2_. Additionally, it had a CO_2_/N_2_ (15/85, V/V) IAST selectivity of 32.8, a breakthrough time of 11.5 min, and 97.6% retention capacity after 5 cycles. HK@BNNS‐PEI′s high adsorption capacity and regeneration efficiency make it a promising candidate for onboard CO_2_ capture. A copper‐based homochiral MOF synthesized from mononuclear Cu^2^⁺ centers and an L‐histidine derivative ((S)‐3‐(1H‐imidazol‐5‐yl)‐2‐(4H‐1,2,4‐triazol‐4‐yl)propanoic acid), which coordinated in a square planar geometry, was reported by Capelo‐Avilés et al. for the separation of CO_2_ from CH_4_.^[^ ^217^ ^]^ The TAMOF‐1 has a 10 Å helicoidal channel with a BET surface area of 980 ± 50 m^2^ g^−1^ and stability in a humid environment. The structural integrity of TAMOF‐1 was also not severely affected when it was exposed to strong H_2_S. It demonstrated a CO_2_ uptake of 3.8 mmol g^−1^ at 1 bar and 298 K. Neutron Diffraction and FTIR were used to discover that the binding of CO_2_ molecules in the channel of TAMOF‐1 was with a weak, non‐directional interaction without any sign of chemisorption. The physisorption‐only binding nature of TAMOF‐1 for CO_2_, high CO_2_/CH_4_ (50:50) IAST selectivity of ≈ 40, and breakthrough time of 16.4 min g^−1^ makes it a robust sorbent. Due to the importance of CO_2_ capture at low pressure, Yadav et al. developed mCBMOF‐1, which is a copper (Cu (II)) paddlewheel‐based MOF with 1D channel, and a 6.042 Å spaced four open metal sites that promoted the strong interaction with NH_3_.^[^ ^218^ ^]^ The presence of NH_3_ in the open metal site increased the CO_2_ uptake by 106% compared to that of the pristine mCBMOF‐1, promoted by the formation of carbamic acid between NH_3_ and CO_2_. It has a type 1 N_2_ isotherm at 77 K and 1 bar and a BET surface area of 996 m^2^ g^−1^ that decreased to 686 m^2^ g^−1^ after exposure to NH_3_. Before the exposure to NH_3_ (at 1 bar, 298 K), it had a low CO_2_ isosteric heat of adsorption (*Q*_st_) of 28 kJ mol^−1^ that increased to 39 kJ mol^−1^ afterwards. The CO_2_ uptake at 298 K and 0.15 bar considered to be important due to the reduced energy penalty jumped from 0.51 mmol g^−1^ for the pristine mCBMOF‐1 to 1.05 mmol g^−1^ for the NH_3_‐doped mCBMOF‐1, corresponding to a 106% increase. This implies that the application of this MOF in onboard carbon capture will not require high pressure for optimal results, thereby saving big of energy. In another work, Hu et al. investigated the capture of CO_2_ directly from flue gas. In the study, four isostructural MOFs, namely TIFSIX‐Cu‐TPA, SIFSIX‐Cu‐TPA, GeFSIX‐Cu‐TPA, and NbOFFIVE‐Cu‐TPA were synthesized.^[^ ^219^ ^]^ The performance of TIFSIX‐Cu‐TPA made from [TiF_6_]^2−^ anionic pillar, Cu^2^⁺ cationic nodes, and Tri(pyridin‐4‐yl)amine organic linker surpassed the others due to the high uptake of CO_2_ to the tune of 2.31 mmol g^−1^ at 0.15 bar (and 298 K) and a total of 4.70 mmol g^−1^ at 1 bar (and 298 K). TIFSIX‐Cu‐TPA is stable in a humid and acidic environment and thermally stable up to 308 °C. The regeneration of this TIFSIX‐Cu‐TPA is energy efficient based on the low heat of adsorption, which is 39.2 kJ mol^−1^. Its robustness after ten regeneration cycles, IAST selectivity (CO_2_/N_2_, 15/85) of 910, and a breakthrough (CO_2_/N_2_, 15/85) time at 298 K of 33.6 min makes it suitable for practical application. The uniqueness of TIFSIX‐Cu‐TPA is because of its dual cage system (small cage ≈4 Å and large cages ≈8.5 Å) that facilitates high uptake capacity and CO_2_/N_2_ selectivity. Chen et al reported another MOF that has the capacity for CO_2_ adsorption at low pressure.^[^ ^220^ ^]^ The scalable MIL‐120(Al)‐AP from Al(OH)(OAc) and H_4_BTeC is a cheap sorbent made from readily available materials and can easily be incorporated into onboard carbon capture. It demonstrated a 1.90 mmol g^−1^ CO_2_ uptake at 0.1 bar and 3.87 mmol g^−1^ at 1 bar, all at 298 K. The crystallinity of the framework was preserved after boiling in water for 10 days, and thermally stable up to 400 °C. The IAST selectivity (15% CO_2_ / 85% N_2_) is greater than 80. The techno‐economic analysis for the industrial production of this MOF shows that it is feasible, costing only ≈$13 kg^−1^ at the kiloton‐scale.

Recent ‘ship‐in‐a‐bottle’ crosslinking of alkylamines with epoxides inside MOF pores (MIL‐101(Cr)) yields MOF–polymer composites with enhanced stability and capacity under relevant conditions. The top performer (Cr‐BDC–TAEA–BDE) shows 2.2 mmol g^−1^ at 0.15 bar, 313 K, CO_2_/N_2_ selectivity of 301, Q_st_ ≈ 110 kJ mol^−1^, and prolonged separation times (≈103 min g^−1^ dry; ≈143 min g^−1^ humid), with minimal capacity loss after 100 TSA cycles. Compared to non‐crosslinked amine‐impregnated MIL‐101(Cr), in‐pore crosslinking suppresses amine leaching and improves cyclic retention, making this strategy well‐suited to humid, low‐grade‐heat maritime operation.^[^ ^221^ ^]^ Although the use of sorbents (**Table** **1**) to capture CO_2_ is considered an energy‐saving process, the sorbents, which are usually in powder or crystalline forms, cannot be used directly without undergoing further engineering processes to pelletize them. This is because of the potential effects of the wave of the sea and air from the exhaust chamber, which can hinder the efficient mounting of the sorbent‐loaded CO_2_ capture system onboard. Majchrzak‐Kucęba and Ściubidło reported the shaping of various MOFs into pellets to achieve these goals using a no‐binder pelletization method.^[^ ^222^ ^]^ In their report, tableting pressure per time was used to compress the structure of CuBTC and MIL‐53(AI) at 3.7, 7.4, 29.6, and 59.2 (kN m^−^^2^) regimes at 0.5 and 2 min intervals, respectively. It was observed that the crystallinity of the MOFs was affected at high pressure. This negatively impacted the CO_2_ uptake capacity of CuBTC compared to the original but had no effect on the capacity of MIL‐53(AI). This implies that although palletization is a direction to consider, there is no single standard condition that will be suitable for all sorbents, and hence has to be treated on a case‐by‐case basis. Additionally, the process of pelletization requires a large amount of sorbent, which is a significant drawback.

**Table 1:** Sorbents for carbon dioxide capture and separation at varied conditions.

Sorbents	Structural Composition	CO_2_ Uptake Capacity#	Temperature [K]	Pressure	Relative Humidity	Refs.
PCN‐250(Fe_3_)	Built from trigonal [Fe_3_(μ_3_‐O) (CH_3_COO) _6_] nodes connected by azobenzenetetracarboxylate (ABTC⁴⁻) linkers into a microporous 3D framework with pore apertures centered ≈ 5.9 Å	1.18 mmol g^−1^ 1.82 mmol g^−1^	298 298	1 bar 1 bar	dry (0%) 50%	[1]
PCN ‐250(Fe_2_Co)	Maintains the same ABTC⁴⁻ linker but substitutes one Fe^2^⁺/Fe^3^⁺ site with Co^2^⁺ in the [Fe_2_Co(μ_3_‐O) (CH_3_COO) _6_] cluster, forming an isostructural framework with PCN‐250(Fe_3_)	1.32 mmol g^−1^ 2.23 mmol g^−1^	298 298	1 bar 1 bar	dry (0%) 50%	[1]
CALF‐20	a zinc‐based MOF constructed from 1,2,4‐triazolate and oxalate linkers, forming a 3D dmc (diamondoid‐like) topology with micropores of ≈ 3–4.3 Å	2.60 mmol g^−1^ 3.30 mmol g^−1^ (simulated) 2.60 mmol g^−1^	298 273 298	0.15 bar (at flue gas partial pressure) 0.15 bar 0.15 bar	dry (0%) dry (0%) 40%	[2]
SIFIX‐3‐Ni	consists of pyrazine‐linked Ni^2^⁺ layers pillared by SiF_6_^2^⁻ anions, creating a cubic framework with 3.8 Å fluorine‐lined pores	≈60 cm^3^ g^−1^ ≈40 cm^3^ g^−1^ (simulated) ≈1.0 mmol g^−1^ ≈60 cm^3^ g^−1^ (after 14 days) ≈109.5 mg g^−1^ CO_2_ (Water Saturated CO_2_ atmosphere) ≈109.5 mg g^−1^ CO_2_ (dry CO_2_ condition)	293 293 293 293 298 298	1 bar 0.15 bar (at flue gas partial pressure) 0.4 mbar (Direct‐air capture condition) 1 bar 1 bar 1 bar	dry (0%) dry (0%) dry (0%) 75% 100% dry (0%)	[3]
COF‐609	an imine‐linked network constructed from 2,4,6‐tris (4‐formylphenyl)‐1,3,5‐triazine (TFPT) and 4,4′‐diaminobenzanilide (DABA) that assembles hexagonal sheets and possesses 1D channel pore dimensions of ≈37 Å	6.8 cm^3^ g^−1^ 29.0 cm^3^ g^−1^	298 298	0.4 mbar 40 mbar (natural gas flue gas)	dry (0%) dry (0%)	[4]
COF‐999	Olefin‐linked amine‐functionalized COF with ethylene‐bridged secondary amines (–CH_2_–CH_2_–NH–) as CO_2_ chemisorption sites	≈0.96 mmol g^−1^ ≈ 1.69 mmol g^−1^ ≈2.05 mmol g^−1^ ≈ 2.09 mmol g^−1^	298 298 298 298	400 ppm 400 ppm 400 ppm 400 ppm	dry (0%) 25% 50% 75%	[5]
Zeolite 13X	a microporous aluminosilicate with a faujasite (FAU) topology, featuring a 3D network of super cages (≈13 Å) connected by 7.4 Å windows	≈180 mg g^−1^ ≈140 mg g^−1^ ≈60 mg g^−1^ ≈ 0 mg g^−1^ (water rapidly occupies Na⁺ sites, displacing CO_2_)	298 298 298 298	1 bar 1 bar 1 bar 1 bar	dry (0%) dry (0%) dry (0%) 100%	[3]
FJI‐H14	zinc‐based MOF constructed from Zn_4_O clusters and 4,4′,4′′‐s‐triazine‐2,4,6‐triyl‐tribenzoate (TATB) linkers, forming a 3D cubic framework with pcu topology that features octahedral and tetrahedral cages (≈7–12 Å) interconnected by triangular windows	7.23 mmol g^−1^ 5.46 mmol g^−1^ 5.30 mmol g^−1^	298 298 298	1 bar 0.15 bar 1 bar	dry (0%) dry (0%) 50% (For 24h)	[6]
MOF‐808‐Lys	a Zr_6_O_4_(OH)_4_ cluster–based framework built from 1,3,5‐benzenetricarboxylate (BTC) linkers, forming a 3D mesoporous structure with cage‐like pores (≈18 Å) interconnected by windows (≈14 Å); its pendant lysine amino acid groups are grafted to the Zr–oxo nodes	0.612 mmol g^−1^ 1.205 mmol g^−1^ (97% increase over dry)	298 298	400 ppm 400 ppm	dry (0%) 50%	[7]
TAMOF‐1	a Cu^2^⁺‐based homochiral MOF constructed from an L‐histidine‐derived ligand, forming a 3D cubic framework with helicoidal, interconnected 10 Å‐wide channels decorated with carboxylate, triazole, and imidazole groups	2.25 mmol g^−1^ 1.73 mmol g^−1^ 1.68 mmol g^−1^ (> 95% retention, Stable under 20 repeated humid adsorption–desorption) 1.55 mmol g^−1^ 0.12 mmol g^−1^ (Active in DAC regime; maintained after multiple cycles)	298 298 298 298 298	1 bar 0.15 bar 0.15 bar 0.15 bar 0.4 bar	dry (0%) dry (0%) 40% 60% 40%	[8]
PPN‐6‐CH_2_DETA	covalently bonded framework synthesized via Yamamoto coupling of tetrakis(4‐bromophenyl) methane, forming a 3D, amorphous network of interconnected micropores (≈1–2 nm) with exceptionally high surface area (up to 4023 m^2^·g^−1^); post synthetic polyamine tethering (e.g., DETA) introduces amine‐functionalized pores	4.3 mmol g^−1^ 3.0 mmol g^−1^ 2.54 mmol g^−1^	298 298 298	1 bar 0.15 bar 0.15 bar	dry (0%) dry (0%) dry (0%)	[9]
PPN‐125‐DETA	amine‐tethered microporous phenolic polymer built from phloroglucinol and terephthalaldehyde, forming a disordered 3D network (≈1–2 nm pores) with covalently grafted polyalkylamine chains	1.4 mmol g^−1^ 0.97 mmol g^−1^ (Working capacity at 120 °C regeneration, > 90% capacity retained)	298 313	0.15 bar 0.15 bar	dry (0%) dry (0%)	[10]

## 7 Amination Strategy as a Tool to Enhance Carbon Capture Capabilities

Executing oceanic carbon capture is complicated and unforgiving controlling for supersaturation of humidity and varying levels of CO_2_, limited device footprint with subsequent interconnectivity and bonding, and the properties of materials exist due to necessary chemical and mechanical stability that must be obtained. Porous frameworks have been some of the more studied for such purposes, including metal‐organic frameworks (MOFs), covalent organic frameworks (COFs), and porous polymer networks (PPNs) because they stabilize the inclusion of aliphatic amines, which is one of the most found classes of CO_2_‐reactive species. This subsection takes note of relevant advancements in amine‐inclined porous frameworks as well as the material construction properties of interest that make these lab achievements more transferable to successful oceanic capture. Relative to other reactive capture means, amines are effective because they′re usually Lewis bases and nucleophiles, which is why so much work has been done to incorporate such species as part of the framework materials. Relative to hydroxides, amines are less Lewis basic and have a less dense population of reactive sites; however, the diversity of possible geometric configurations is the driving force of effectiveness.

The reaction pathway for CO_2_ capture typically involves the generation of carbamate or carbamic acid species through a reversible process. The reaction selectivity and efficiency are largely dependent on the amine structure: primary and secondary amines are considerably more reactive, and tertiary amines tend to fail for CO_2_ capture in anhydrous conditions. In addition, primary amines have lower steric hindrance and benefit from much faster reaction kinetics to effectively capture CO_2_. Therefore, amines contained in the pore architectures of MOFs, COFs, and PPNs are a viable, easily scalable solution to liquid absorbent failures and promote the necessary use of carbon capture at sea.

**Figure 3:**
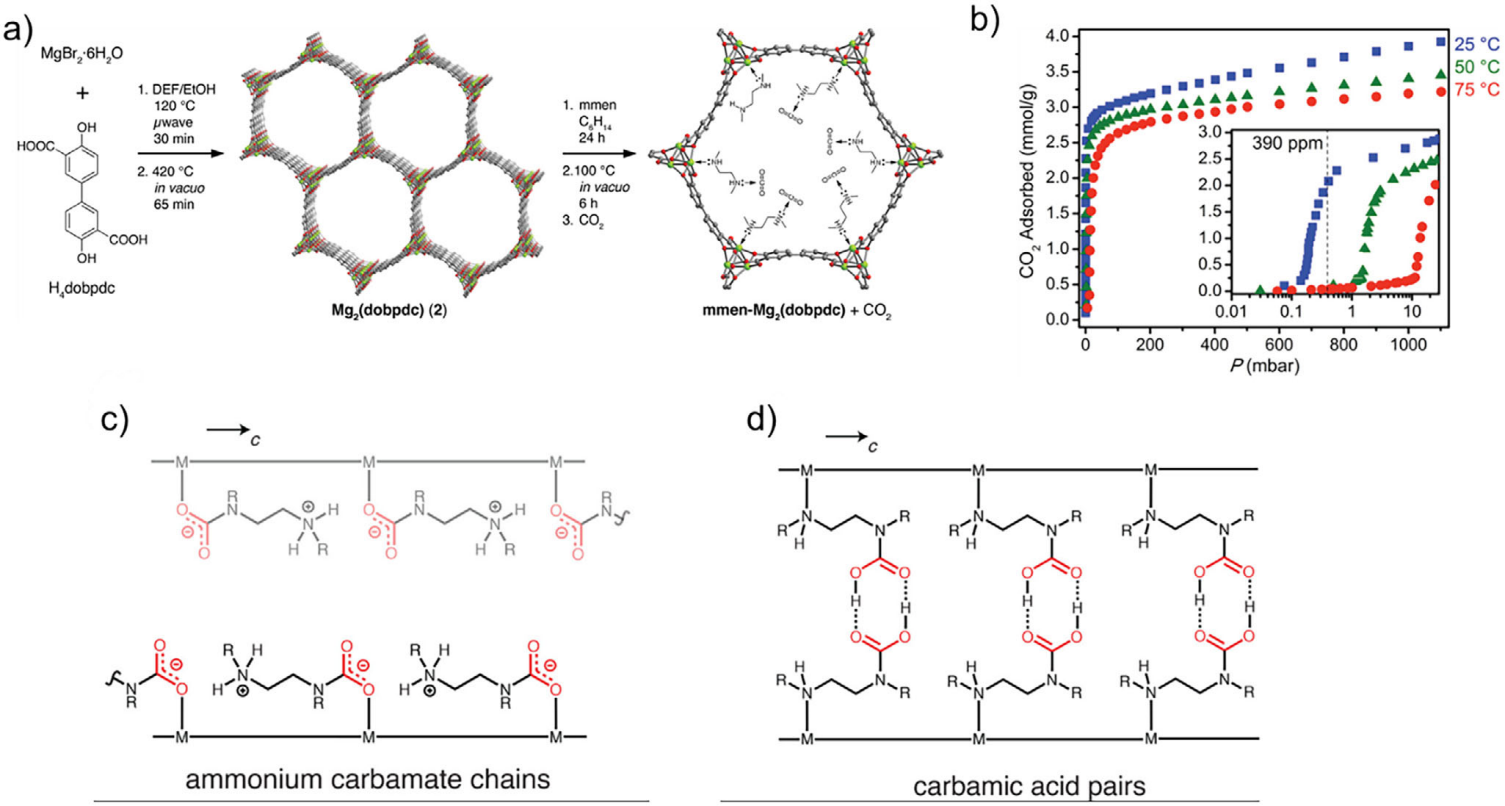
a) Synthesis and structure of Mg_2_(dobpdc) and mmen‐Mg_2_(dobpdc) (mmen = N, N′‐dimethylethylene diamine; dobpdc4− = 4,4′‐dioxido‐3,3′‐biphenyldicarboxylate) (b) Adsorption of CO_2_ in mmen‐Mg_2_(dobpdc) at 25 °C. Reproduced with permission.^[^ ^120^ ^]^ Copyright 2012, American Chemical Society. c,d) Schematic illustration of Ammonium carbamate chain and carbamic acid pair formation upon CO_2_ adsorption, respectively. Reproduced with permission.^[^ ^115^ ^]^ Copyright 2018, American Chemical Society.

Baron et al. reported the first major study of amine‐functionalized MIL‐53(Al), based on infinite chains of corner‐sharing AlO_4_(OH)_2_ octahedra connected by 2‐aminoterephthalate linkers to form a diamond‐shaped, flexible, 1D channel structure.^[^ ^119^ ^]^ Functionalizing MIL‐53 with amino groups dramatically enhanced CO_2_ selectivity, achieving a CO_2_/CH_4_ separation factor greater than 60 under low surface coverage. The amino‐functionalized pores increased the interaction strength between CO_2_ and the framework compared to the pristine MIL‐53(Al), which itself exhibits a well‐known breathing behavior upon gas adsorption. In general, amines featuring longer N‐substituted alkyl chains tend to display enhanced Lewis basicity, while the presence of multiple amino groups along a single alkyl chain increases the density of chemisorption sites within MOF structures. In this regard, McDonald, Long, and co‐workers pioneered the functionalization of Mg_2_(dobpdc), an expanded MOF‐74 analogue with 18.4 Å diameter hexagonal channels lined by open Mg^2^⁺ coordination sites, using N, N′‐dimethylethylenediamine (mmen).^[^ ^120^ ^]^ (**Figure** **3**a) The resulting mmen–Mg_2_(dobpdc) material displayed outstanding CO_2_ capture capacity under low‐pressure conditions relevant to both direct air capture (DAC) and flue gas treatment. The material adsorbed 2.0 mmol g^−1^ at 390 ppm CO_2_ (25 °C) and 3.14 mmol g^−1^ at 0.15 bar CO_2_ (40 °C), (Figure 3b) with rapid cycling and minimal capacity loss over repeated adsorption‐desorption cycles. The heat of CO_2_ adsorption was measured at −71 kJ mol^−1^, enabling effective regeneration at low energy costs (≈2.34 MJ kg^−1^ CO_2_). The highly open and modular pore architecture of Mg_2_(dobpdc), combined with stable alkylamine grafting, makes this material particularly attractive for maritime CO_2_ capture applications, where fast kinetics, high selectivity under low CO_2_ partial pressures, and low‐energy regeneration are critically important. In another work, Forse et al. (2018) systematically characterized diamine–M_2_(dobpdc) MOFs (M = Mg, Mn, Fe, Co, Ni, Zn). These frameworks have hexagonal 1D channels and modular coordination sites, ideal for controlled amine functionalization. Using multinuclear NMR and DFT, the authors showed that most variants form ammonium carbamate chains, while dmpn–Mg_2_(dobpdc) uniquely displays a mixed chemisorption mechanism of carbamate and carbamic acid species (Figure 3c,d).^[^ ^115^ ^]^

**Figure 4:**
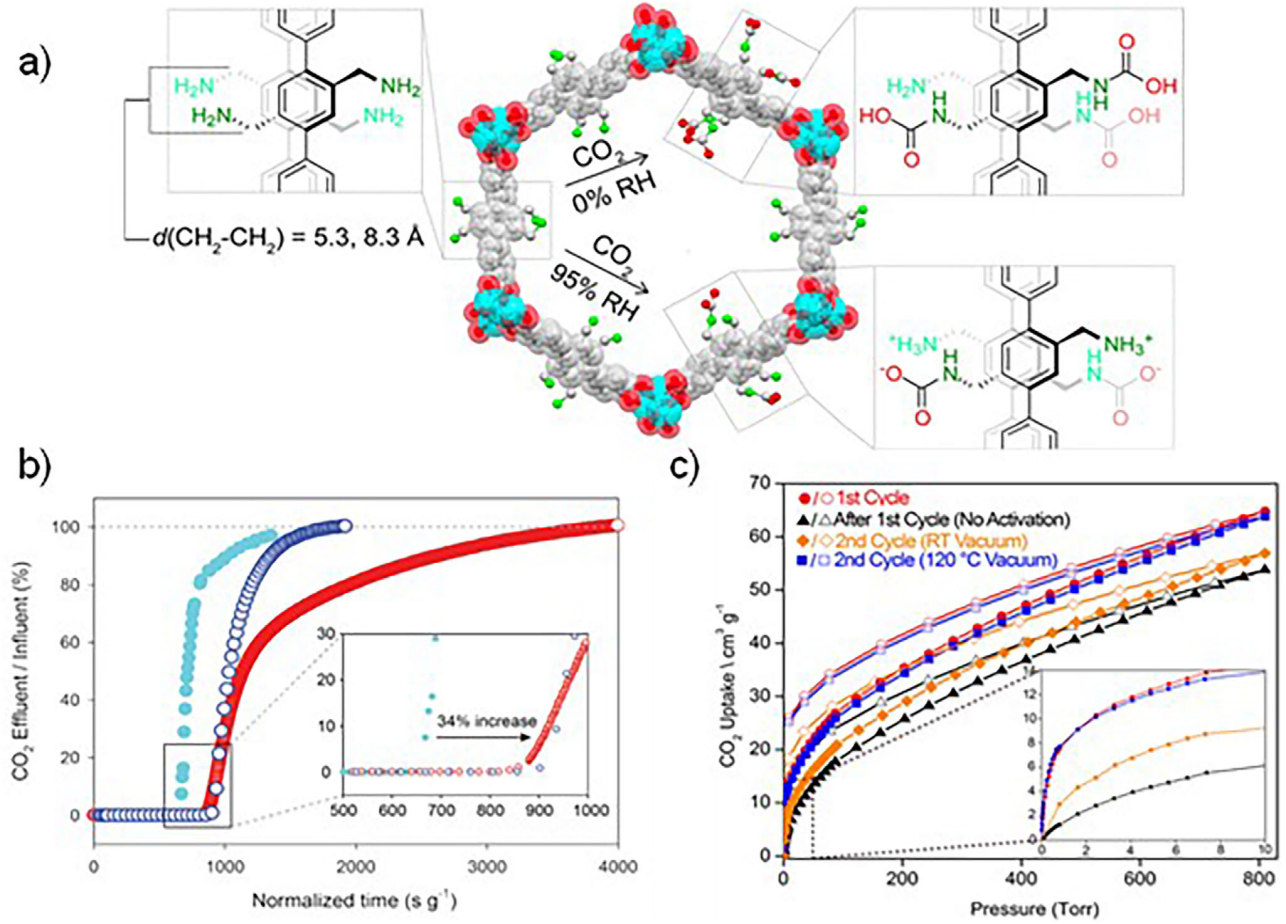
a) View of modeled IRMOF‐74‐III‐(CH_2_NH_2_)_2_, depicting the three possible pore environments before (left pore wall) and after exposure to CO_2_ under 95% RH (bottom right pore wall) and dry (upper right pore wall) conditions. b) Breakthrough curves for IRMOF‐74‐III‐(CH_2_NH_2_)_2_ under dry conditions (dark blue empty markers) and wet conditions (red empty markers), and for comparison, IRMOF‐74‐IIICH_2_NH_2_ under wet conditions (cyan filled markers). c) CO_2_ isotherms (closed symbols = adsorption; open symbols = desorption) for IRMOF‐74‐III‐(CH_2_NH_2_)_2_ at different conditions. Reproduced with permission.^[^ ^98^ ^]^ Copyright 2017, American Chemical Society.

Yaghi and co‐workers reported a diamine‐functionalized metal–organic framework, IRMOF‐74‐III‐(CH_2_NH_2_)_2_, exhibits highly efficient CO_2_ capture through chemisorption, forming carbamic acid in dry conditions and shifting to ammonium carbamate in the presence of water vapor.^[^ ^98^ ^]^ The MOF features large 1D hexagonal channels with alkylamine groups covalently tethered to the linkers, allowing for accessible and well‐positioned reactive sites. Structural modeling revealed amine–amine distances (5.3 and 8.3 Å) (**Figure** **4**a) that are geometrically favorable for carbamic acid formation, while water mediates proton transfer to favor ammonium carbamate under humid conditions. At low pressure (<100 Torr), it outperforms its monoamine analog by a factor of 2.33, showing enhanced CO_2_ uptake (1.2 mmol g^−1^) and desorption at ≈47 °C. Under 95% RH, the same uptake is maintained, but with stronger binding and desorption shifting to ≈65 °C, notably, the MOF retains structural integrity and adsorption capacity across cycles, with full regeneration achieved at 120 °C (Figure 4b,c). Its resilience under humid conditions, low‐temperature regeneration, and high selectivity make it a strong candidate for maritime carbon capture, where systems must operate in moisture‐rich exhaust streams and under energy‐efficient conditions.

**Figure 5:**
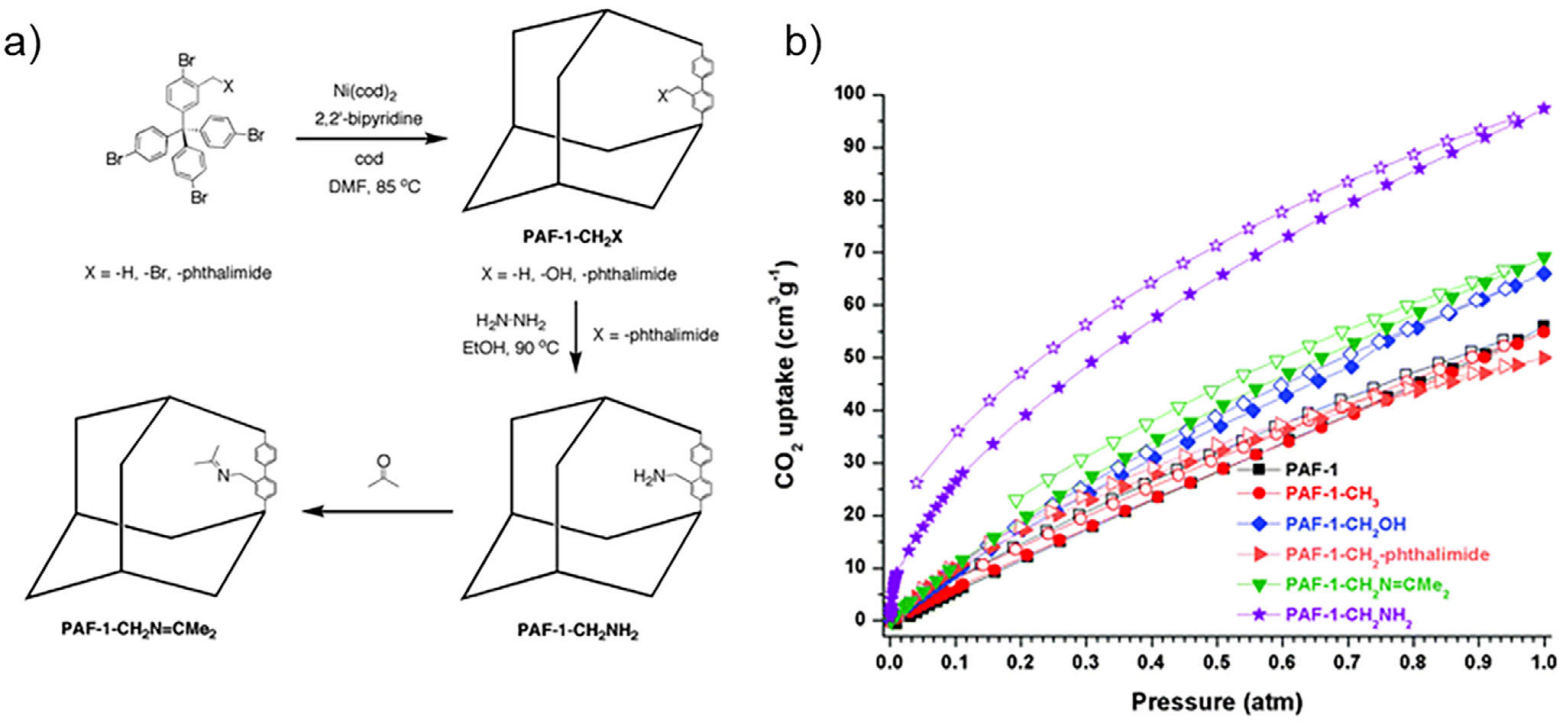
a) the synthesis and structure of PAF‐1–CH_2_X (X = H, OH, NH_2_) and PAF‐1–CH_2_–phthalimide b) CO_2_ isotherms for **PAF‐1** derivatives measured at 273 K. Closed symbols = adsorption; open symbols = desorption. Reproduced with permission.^[^ ^123^ ^]^ Copyright 2013, Royal Society of Chemistry.

While much attention has been given to amine‐functionalized MOFs, amine tethering has also proven highly effective in enhancing CO_2_ capture in alternative porous sorbents such as porous polymer networks (PPNs). These materials offer complementary advantages for maritime CO_2_ capture, including exceptional chemical stability, high amine loading, and tunable pore architectures. Lu et al. reported polyamine‐tethered porous polymer networks (PPN‐6) based on a rigid biphenyl framework with extremely high surface area (4023 m^2^ g^−1^).^[^ ^122^ ^]^ PPN‐6 was modified with various polyamines, including diethylenetriamine (PPN‐6‐CH_2_DETA), (**Figure** **5**a) which achieved a remarkable CO_2_ uptake of 4.3 mmol g^−1^ (15.8 wt%) at 295 K and 1 bar. At more realistic post‐combustion flue gas conditions (0.15 bar CO_2_), PPN‐6‐CH_2_DETA maintained 3.0 mmol g^−1^ CO_2_ capacity with minimal N_2_ uptake, leading to exceptional CO_2_/N_2_ selectivity. The all‐covalent network of PPN‐6 enhances its chemical and thermal stability compared to MOFs, providing an advantage under the fluctuating temperatures, pressures, and humid conditions characteristic of maritime CO_2_ capture.

In another work, Lu et al. developed a more scalable and low‐cost synthesis for amine‐tethered PPNs, introducing PPN‐125‐DETA.^[^ ^116^ ^]^ This phenolic resin‐type porous polymer network (Figure 5c) (BET area ≈702 m^2^ g^−1^) was functionalized with diethylenetriamine, dramatically increasing CO_2_ uptake to 1.4 mmol g^−1^ at 0.15 bar and 25 °C. PPN‐125‐DETA displayed superior energy efficiency: regeneration energy was ≈3156 kJ kg^−1^ CO_2_, more than three times lower than for traditional aqueous monoethanolamine (MEA) solutions. The material retained over 90% of its capacity after 50 adsorption–desorption cycles under temperature swing conditions.

Another achievement in the porous polymeric materials development for CO_2_ capture is the implementation by Garibay et al. They developed a new generation of functionalized porous aromatic frameworks (PAFs), with PAF‐1 derivatives studied the most. The structure of PAF‐1 consists of tetrahedral tetrakis(4‐bromophenyl) methane units with a rigid, non‐catenated diamondoid topology and an ultra‐high surface area (≈4100 m^2^ g^−1^).^[^ ^123^ ^]^ The authors anchored methyl, hydroxymethyl and aminomethyl groups onto the PAF‐1 backbone via post‐synthetic modification (PSM) and de novo synthesis. (Figure 5a) Among the derivatives, the amine functionalized PAF‐1–CH_2_NH_2_, exhibited exceptional CO_2_ uptake (98 cm^3^ g^−1^ at 1 bar and 273 K), ≈2 times larger than the pristine PAF‐1. (Figure 5b) This strong interaction with CO_2_ can be attributed to its high isosteric heat of adsorption (≈57.6 kJ mol^−1^). Furthermore, PAF‐1–CH_2_NH_2_ is thermally stable between 300–400 °C and hydrophobic, indicating appropriateness for CO_2_ sequestration.

Amine functionalization has emerged as a successful strategy not only in MOFs and PPNs but also in covalent organic frameworks (COFs) to facilitate CO_2_ chemisorption. The advantages of using COFs for an at‐sea application include their crystalline porous morphology with chemical and thermal stability, adjustable pore sizes, and stability in a humid, fluctuating atmosphere, ideal for in‐hull scrubbing agents on Navy vessels.

**Figure 6:**
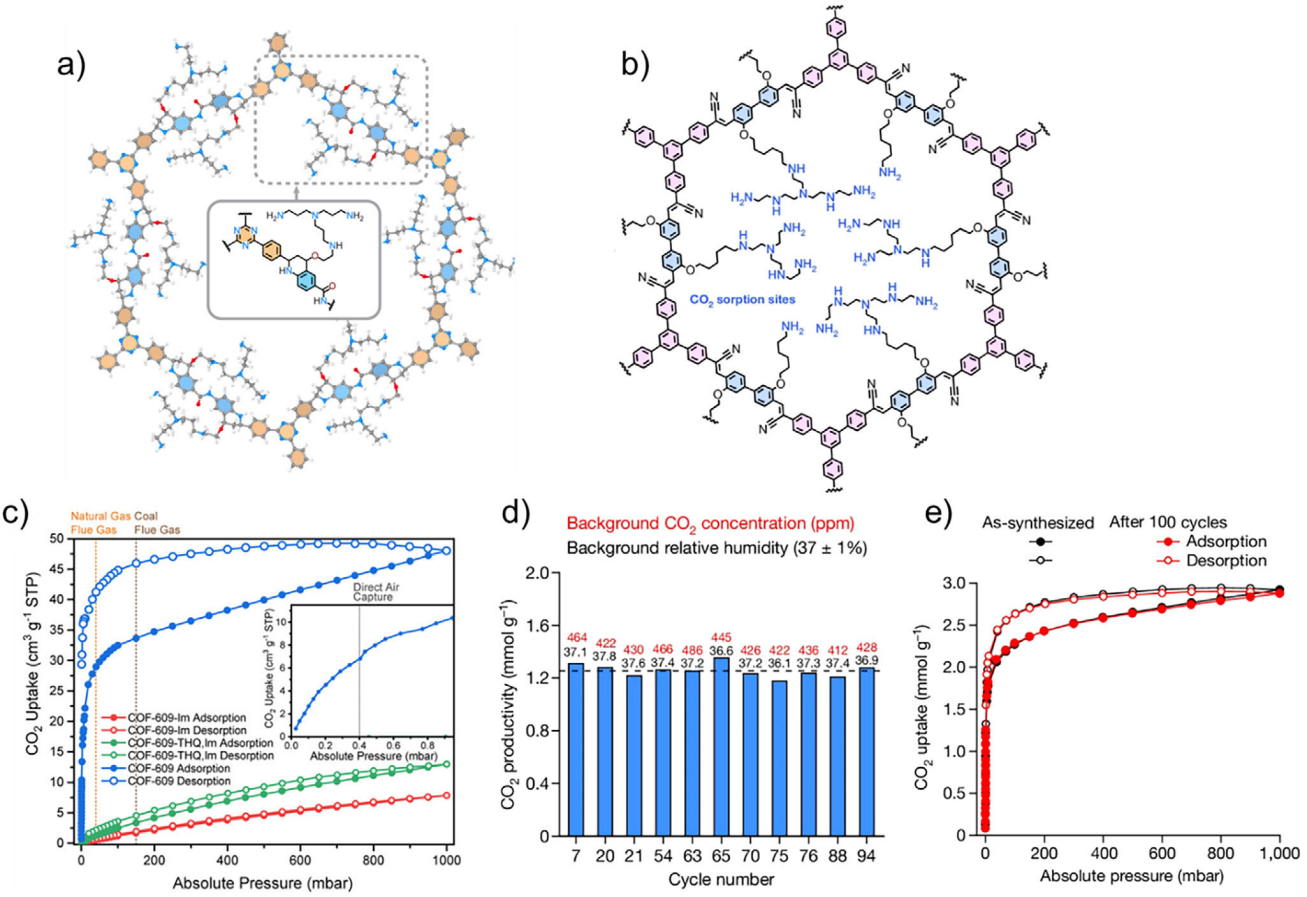
a) structure of COF‐609 (b) single‐component CO_2_ isotherms (25 °C) of COF‐609‐Im, COF‐609‐THQ, Im, and COF‐609. The inset in panel (b) displays a zoomed‐in view of the adsorption branch of COF‐609 at 0–1 mbar to highlight the uptake at the DAC‐relevant pressure. Reproduced with permission.^[^ ^124^ ^]^ Copyright 2022, American Chemical Society. c) structure of COF‐999 (d) CO_2_ productivity of selected cycles with an ambient RH of 37 ± 1%, with corresponding ambient outdoor CO_2_ concentration. The average productivity of selected cycles is shown as the dotted line (e) Comparison of single‐component CO_2_ sorption isotherms (25 °C) of as‐synthesized COF‐999 and the sample after 100 outdoor air cycles. Reproduced with permission.[30] Copyright 2024, Springer Nature.

A great example of reactive aliphatic amines covalently bonded into COFs for subsequent CO_2_ adsorption was reported by Yaghi and Co‐workers.^[^ ^124^ ^]^ They generated COF‐609‐Im, an imine‐linked network constructed from 2,4,6‐tris(4‐formylphenyl)‐1,3,5‐triazine (TFPT) and 4,4′‐diaminobenzanilide (DABA) that assembles hexagonal sheets and possesses 1D channel pore dimensions of ≈37 Å in diameter per PXRD and N_2_ sorption (BET surface area ≈724 m^2^ g^−1^). Then, the imine linkages were partially transformed into base‐stable tetrahydroquinoline (THQ) linkages through aza‐Diels−Alder cycloaddition, followed by covalent grafting of tris(3‐aminopropyl) amine (TRPN). This unprecedented transformation maintained the integrity of the framework structure but with a covalent change to grafted amines at a high loading density within the pore walls (**Figure** **6**a). COF‐609 exhibited a 1360‐fold increase in CO_2_ absorption relative to the unmodified COF (0.304 mmol g^−1^ vs. 0.00022 mmol g^−1^ at 0.4 mbar) (Figure 6b) and an extra 29% at 50% relative humidity, ideal for capturing marine exhaust systems because of the aqueous environments. The binding mechanism occurred via carbamate formation, the more favorable binding mode in the presence of water. Thus, while the modification created a loss of crystallinity, chemical stability and access to pores remained, indicating that this sorbent is an ideal candidate for humid capture systems since solid sorbents must be stable and regenerable, as well as water tolerant.

Yaghi et al. (2024) have revealed a crystalline olefin‐linked COF, COF‐999, designed for direct air capture under realistic conditions.^[^ ^30^ ^]^ COF‐999 was formed through Knoevenagel condensation of BPDA‐N_3_ and TCPB, resulting in a honeycomb structure with AA stacking, mesopores of 3.3 nm. (Figure 6c). A post‐synthetic functionalization proceeded as well: azides were first converted to amines, then polyamines through aziridine to afford COF‐999 with considerable polyamine loadings stably grafted in situ within the pores. The resultant material was effective: CO_2_ uptakes measured 0.96 mmol g^−1^ from dry air and 2.05 mmol g^−1^ at 50% relative humidity (both at 400 ppm CO_2_). Most impressively, COF‐999 achieved full working capacity after 100 temperature‐swing adsorption‐desorption tests in open air, tested in Berkeley, CA. (Figure 6d,e). Furthermore, it achieved ultrafast kinetics for CO_2_ adsorption, reaching half its capacity in less than 19 min in ambient air and could be regenerated at a low temperature of 60 °C, an energy‐efficient condition ideal for small‐batch capture systems made for ships.

## 8 Conclusion

Porous material‐based, such as Metal‐Organic Framework (MOFs) for carbon capture systems (CCS), represent an innovative and technically feasible pathway to decarbonize maritime transport, particularly during the transitional period before full‐scale alternative fuels and propulsion systems become mainstream. Their potential lies in their modularity, compatibility with waste heat utilization, and capacity for retrofitting. However, practical deployment remains constrained by material performance under marine conditions, the spatial and operational dynamics of ships, and the prohibitive costs associated with installation, maintenance, and operation.

Addressing these setbacks requires a multi‐pronged approach. Research into more robust, marine‐compatible porous materials, such as Metal‐Organic Framework (MOFs) with high selectivity and rapid regeneration, is essential. Process intensification strategies, such as integrating adsorption columns with hybrid systems (e.g., cryogenic or membrane stages), could improve efficiency and reduce the footprint. Regulatory frameworks must evolve to recognize and reward CCS contributions to emissions reductions, while port authorities and international agencies must coordinate to build the required CO_2_‐handling infrastructure. Ultimately, porous‐material‐based onboard carbon capture and storage (OCCS) systems to play a central role in maritime decarbonization, they must move from academic and pilot‐scale demonstrations into scalable and commercially attractive technologies. This will only happen through coordinated innovation, policy support, and cross‐sector collaboration between material scientists, marine engineers, regulators, and ship operators.

Integration concerns from limited space and dynamic operation complicate how these systems would function onboard vessels, as effective systems must be small, durable, and easy to use. Therefore, new capture systems must be proposed that require small adsorption units; hybrid systems that work with other air‐handling systems should be explored. Techno‐economics is another critical barrier. Capital and operational costs are exorbitantly high to the point where ship operators will not use such technology without external regulatory pressure or incentives. Improved efficiency for materials, better energy use, and focusing on more straightforward reactivation/regeneration processes can all benefit costs over the material′s lifetime.

Regulations and infrastructure also need to accompany successful capture integration to ensure appropriate management of captured carbon dioxide (CO_2_). Without global regulations detailing how captured CO_2_ will be used, and port‐related infrastructure to tell those aboard how it will be dealt with, widespread integration will fail. International regulations must be created, and ports must be enhanced to accept and store released CO_2_. Ultimately, improvements for maritime carbon capture rely on research and established developments, industrial applications, governmental protocols, and international shipping councils to create harmony that reduces the red tape in the end, yet fosters effective implementation across vessels. Addressing these improvements alone only causes future commercial applications and global decarbonization hurdles down the line.

Looking ahead, the advancement of data‐driven design, molecular simulations, and high‐throughput screening will accelerate the identification of new sorbents optimized for marine conditions. Integrating AI‐assisted material discovery with experimental validation can shorten development cycles and identify promising materials tailored for onboard systems. Additionally, coupling OCCS with carbon utilization strategies—such as onboard methanol synthesis or mineral carbonation—could transform captured CO_2_ into valuable products, improving system economics. These synergistic pathways will define the next generation of maritime decarbonization, ensuring that the shipping industry not only captures but also reuses its emissions for value‐added products and hence contributes to the circular economy.

## Conflict of Interest

The authors declare no conflict of interest.
